# Two-Dimensional Cartesian Coordinate System Educational Toolkit: 2D-CACSET

**DOI:** 10.3390/s21186304

**Published:** 2021-09-21

**Authors:** Víctor H. Castañeda-Miranda, Luis F. Luque-Vega, Emmanuel Lopez-Neri, Jesús Antonio Nava-Pintor, Héctor A. Guerrero-Osuna, Gerardo Ornelas-Vargas

**Affiliations:** 1 Posgrado en Ingeniería y Tecnología Aplicada, Universidad Autónoma de Zacatecas, Zacatecas 98000, Mexico; vhcast@uaz.edu.mx (V.H.C.-M.); jesus.nava@uaz.edu.mx (J.A.N.-P.); hectorguerreroo@uaz.edu.mx (H.A.G.-O.); ornelas@uaz.edu.mx (G.O.-V.); 2 Centro de Investigación, Innovación y Desarrollo Tecnológico CIIDETEC-UVM, Universidad del Valle de México, Tlaquepaque 45601, Mexico; emmanuel.lopezne@uvmnet.edu; 3 Unidad Académica de Ingeniería Eléctrica, Universidad Autónoma de Zacatecas, Zacatecas 98000, Mexico

**Keywords:** educational mechatronics, engineering education, hands-on learning, STEM

## Abstract

Engineering education benefits from the application of modern technology, allowing students to learn essential Science, Technology, Engineering, and Mathematics (STEM) related concepts through hands-on experiences. Robotic kits have been used as an innovative tool in some educational fields, being readily accepted and adopted. However, most of the time, such kits’ knowledge level requires understanding basic concepts that are not always appropriate for the student. A critical concept in engineering is the Cartesian Coordinate System (CCS), an essential tool for every engineering, from graphing functions to data analysis in robotics and control applications and beyond. This paper presents the design and implementation of a novel Two-Dimensional Cartesian Coordinate System Educational Toolkit (2D-CACSET) to teach the two-dimensional representations as the first step to construct spatial thinking. This innovative educational toolkit is based on real-time location systems using Ultra-Wide Band technology. It comprises a workbench, four Anchors pinpointing X+, X−, Y+, Y− axes, seven Tags representing points in the plane, one listener connected to a PC collecting the position of the Tags, and a Graphical User Interface displaying these positions. The Educational Mechatronics Conceptual Framework (EMCF) enables constructing knowledge in concrete, graphic, and abstract levels. Hence, the students acquire this knowledge to apply it further down their career path. For this paper, three instructional designs were designed using the 2D-CACSET and the EMCF to learn about coordinate axes, quadrants, and a point in the CCS.

## 1. Introduction

Educational systems are constantly searching for new educational technologies that improve their students’ knowledge and help them acquire the necessary skills in the new industrial era. Innovative classrooms that integrate new information technologies—transforming everyday classrooms into laboratories to improve the students’ academic performance [1,2,3]—are increasingly more common in the teaching–learning process. Due to Coronavirus Disease 19 (COVID-19) distancing restrictions, innovative classrooms are a viable alternative to carry out the practices from home out of the school lab. Education has traditionally involved students interacting with an artifacts in a laboratory, many of them obsolete. Amir H. Behzadan et al. [4] reviewed the effectiveness of technology in the classroom using modern technology through the application of a framework for the visualization of augmented reality in an engineering education context. Their findings indicate that technology is important in student performance when implemented correctly, stimulates greater interaction between the instructor and the student, and encourages cooperative learning, collaboration, problem-solving, and communication skills.

Integrating sensors in educational settings has been correlated with the emerge of digital learning. In [5], four cases where sensors can be integrated to improve learners’ experience and to assess learning retention are analyzed. These cases are sensing technology into the outside environment, sensors for estimating the accuracy of learning retention in the computer classroom, sensing technology applied to E-book reading activities, and sensors to analyze personal emotions in a learning environment. In [6], Computational Thinking was integrated into the middle school science curriculum through a series of data gathering and analysis practices enabled by custom sensors and storylines that engaged the learners. In [7], accelerometers and Artificial Neural Networks were used to improve Physical Education skills.

In [8], a Lab-in-a-box for STEM classes is presented. They integrate a series of sensors, embedded systems, and cloud services to create a low-cost, battery-powered, and internet-connected support material to engage learners in data gathering, data analysis, data representation, and solution making based on information. In [9], sensors were integrated into the classroom to monitor teacher–student interactions in space to create feedback interfaces for learners and educators, assess pedagogical activity for space optimization, and finally accelerate classroom observation analysis cycles. In [10], an air quality kit with Arduino and sensors was developed for students to learn to program and extract patterns from their collected data. In [11], the design and implementation of a weather station for engineering education, as well as an Educational Mechatronics Conceptual Framework, are presented.

Furthermore, as shown in [12], this kind of development enables the transformation of traditional classrooms into an active learning environment without the need to invest in laboratory infrastructure.

This work presents a novel educational toolkit for engineering education, the 2D-CACSET. It involves sensors, embedded systems, cloud computing, and instructional design based on the EMCF to enhance the engineering student learning experience. It is relevant to mention that this educational toolkit can be used as the base to construct other mechatronics concepts related to autonomous robot vehicles control as positioning, linear and angular displacements, kinematics, dynamics, path planning, and control.

## 2. Related Work

The Cartesian coordinate system (CCS) is an abstract concept used in robotics to describe multiple applications and operations. It is of utmost importance for STEM education to ensure that students understand and apply it. The student is usually taught using two-dimensional maps and is invited to perform exercises such as placing points on graphs formed by axes and exercises to calculate and draw lines on this plane, developing mathematical thinking in most cases. However, students are being taught to repeat concepts and solution patterns, without developing spatial thinking, which is highly required in robotics [13,14]. With the current advancement of technology, several strategies can improve teaching of this type of abstract concepts. For example, the use of augmented reality [15], since they involve multiple human sensorimotor processes (spatial thinking) that allow the student to develop a better understanding and learning of abstract concepts [16]. However, the instructional design is still oriented to learn memory concepts but not to understand the origin of the concept so that the student can understand how he came to this knowledge.

Some educational kits have been developed to implement new teaching-learning strategies regarding the Cartesian coordinate system. In [17], a solution is presented with an IMU with accelerometer, gyroscope, and magnetometer in the three axes. However, this solution only offers pure translation; if the participant does not make the indicated movement, the accelerometers will lose the position calculation, so it is very restrictive. In terms of rotation, it works perfectly. In [18], an application with augmented reality in 2D and 3D is presented; it is fascinating as a viewer; however, it lacks an educational methodology to build learning. In [19], they use a drone to develop the course by giving movement instructions, an ideal scenario as a STEM course to enhance spatial thinking; however, they do not present the construction of mathematical concepts (abstract level).

Industry 4.0 challenges the student to face scenarios not typically found in teaching classrooms, manuals, or books. This characteristic requires developing a deep understanding of concepts that can be seen as very easy in the beginning, such as the Cartesian plane [20], and deep management of cognitive operations such as coordination, integration, and structure of the mechatronic concepts. Therefore, a methodology is required to start developing the concept of the Cartesian coordinate system in a spatial context level [13,14], so they can develop spatial thinking. Most studies linking spatial abilities and STEM education have focused on what is termed spatial visualization, which is the process of apprehending, encoding, and mentally manipulating three-dimensional spatial forms. Some spatial visualization tasks involve relating two-dimensional representations to three-dimensional representations and vice versa [21,22]. Then, for simplicity, the two-dimensional representations are considered as the first step for the student to gradually advance in the concept’s construction until the concept is understood in a formal or mathematical description.

## 3. Educational Mechatronics Conceptual Framework EMCF

The EMCF [23,24] will be used in this proposal for the development of mechatronic concepts, particularly the case of the Cartesian plane, also developing spatial thinking [21,22] critical and scientific thinking, known as higher-order thinking [25,26], and finally, the development of mechatronic thinking.

**Figure 1:**
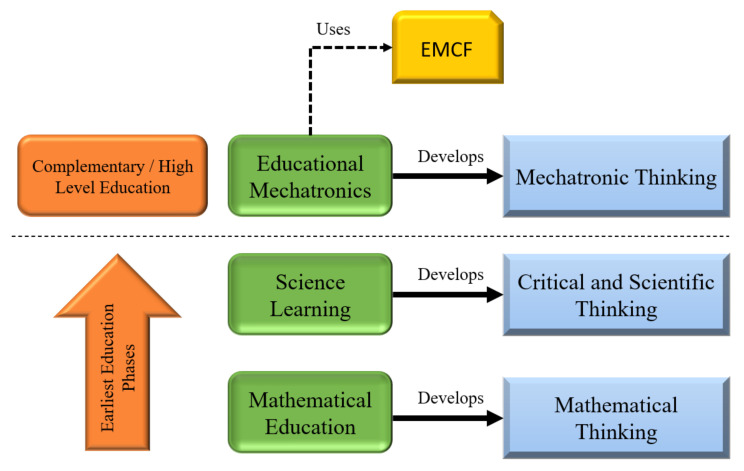
Educative phases.

This framework enables students to appropriate mechatronic concepts through a methodology named Educational Mechatronics. Educational Mechatronics enable the student to learn starting from the concrete learning level (sensorimotor process) to the abstract learning level (mathematical and formal concepts), developing a mechatronic thinking [23,24]. Figure 1 shows the educational phases and their corresponding type of thinking that is developed. Elementary-level education focuses on developing critical, scientific, and mathematical thinking through scientific learning and mathematical education. In high-level education (university) or complementary education, mechatronic thinking could be developed. Is at this level where the EMCF could be applied as a tool for teachers. It can help with the design, implementation, and evaluation of pedagogical activities to develop the mechatronic thinking in students, flexibly and gradually. The EMCF considers connecting spatial thinking and mathematical thinking, thus facing the speed of growth and exponential change of industry 4.0 and responding to the megatrends of the manufacturing industry and advanced manufacturing processes. It focuses on the development, application, or integration of a set of enablers and technologies to generate impact.

**Figure 2:**
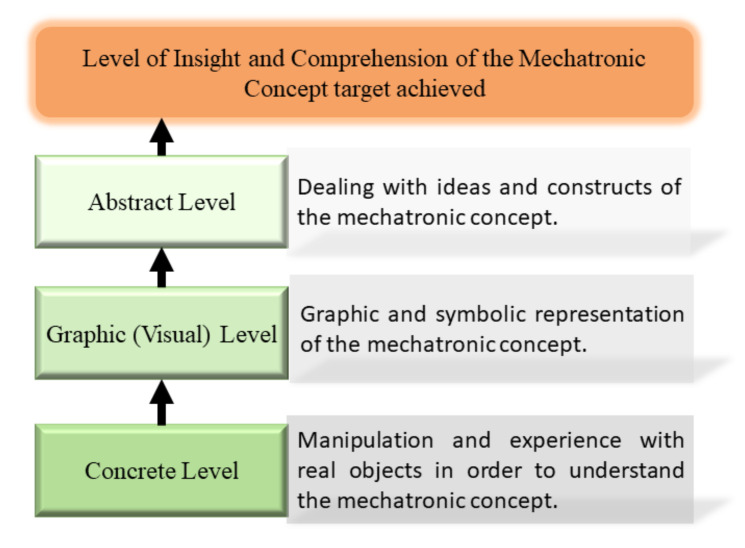
Macro-process of the EMCF Learning Construction Methodology.

The EMCF Learning Construction Methodology (EMCF-LCM) is a macro-process based on a structured teaching methodology [23,24], and it is shown in Figure 2. This EMCF-LCM comprises three learning levels: the concrete, the graphic, and the abstract levels. The concrete level is related to manipulation and experience with real objects, that is, learning focused on the student’s experience with situations of their reality or specific objects. The graphic level represents reality (concrete level) elements with graphics or symbolic elements, enabling students to integrate this knowledge as a skill. The abstract level is totally focused on learning outside reality; this is the greatest abstraction level.

## 4. 2D-CACSET Two-Dimensional Cartesian Coordinate System Educational Toolkit

The 2D and 3D Cartesian coordinates system represent an essential tool for most applied disciplines that deal with engineering, computer graphics, and computer-aided geometric design.

Several systems can be used as an educational tool for the teaching and learning process when dealing with the 2D and 3D Cartesian coordinates. As a design requirement, the educational kit must be wireless since it is planned to be used indoor and outdoor, and to be mounted in different mechatronic prototypes such as mobile robots, drones, and manipulators. Examples of such systems are monocular and stereo vision, motion capture, and real-time location systems (RTLS). RTLS, in particular, involves different technologies like Global Positioning System (GPS), Bluetooth Low Energy (BLE), Wireless Fidelity (WiFi), Zigbee, Infrared (IR), and Ultra-Wide Band (UWB). Therefore, it is necessary to evaluate the differences and advantages to determine which is suitable for our purpose. G. Oguntala et al. [27] developed a performance evaluation of technologies in RTLS applications, which include Wi-FI, Bluetooth, radio frequency identification (RFID), ultrasound, Zigbee, and IR. Using scalability, accuracy, complexity, robustness, energy efficiency, cost, and reliability as metrics, they assigned a score to evaluate feasible points for hybridization, possible challenges and drawbacks, and possible application areas. Their findings indicate that none of these technologies satisfy the performance requirements of any RTLS application since each technology exhibits certain limitations. Consequently, it is application dependent.

There is plenty of information regarding the technology involved in RTLS, which can perform positioning, that is, the process of determining a position of a target. Since we seek to have a flexible educational positioning tool, an RTLS can be helpful for our purpose. RTLS can be classified into indoor and outdoor environments [28].

GPS is a popular solution for the outdoors; however, it is not the recommended technology for indoor applications since satellite radio signals cannot propagate through some structures [29].

For indoor environments, there are different technologies capable of deploying an indoor RTLS. For indoor location systems, one technology used is BLE. For example, [30] develop an RTLS to track employees’ movements in a workplace using BLE beacons and a smartphone as a Gateway. Beacons are placed in different parts of the workplace and exchange messages with the smartphone; this way, the smartphone position is calculated through Received Signal Strength Indicator (RSSI).

Moreoever, IR technology has been proposed as an RTLS solution; in [31] propose the deployment of a real-time location system for inventory control by exchanging codified messages between LEDs and infrared receivers. In addition, in [32] an indoor positioning system is proposed through the hybridization of the built-in Inertial Measurement Unit (IMU), WIFI, and BLE. The user’s walking direction can be estimated, the walking length inferred, and the steps detected, all using a smartphone’s built-in IMU. Furthermore, the authors developed an RTLS using WiFi routers over an established network. Using RSSI, an algorithm is applied to obtain the current location; the result is merged with IMU estimation to avoid drift error. However, the drift problem can occur in some areas where WiFi coverage is inadequate; the authors add an iBeacon BLE device to avoid drift in this case.

Zigbee is another radio frequency (RF) module that can be applied for RTLS. In [33], a network based on Zigbee was deployed, and an algorithm based on RSSI was developed. For proper operation, at least three devices are required. For calculating the target location, each Zigbee device has a known position and sends a signal the moving device reads all and, based on RSSI, sets a radius to draw a circle around each Zigbee device. The location of the target is estimated at the center of the intersecting area of the circles.

UWB is one of the most promising technologies for Real-Time Positioning Systems. State of the art in UWB technology focuses on real-time location systems due to its robustness and precision. According to some studies, massive growth in practical applications with UWB technologies applied to the monitoring of people is expected. In [34], an educational tool is developed to train engineers in the use of UWB technology; the tool allows to apply the acquired knowledge in applications for people location. It is worthwhile to mention that BLE and ZigBee protocols consume less power as compared with UWB, but the last one offers higher precision in a RTLS application [35].

Our flexible educational positioning tool has had several requirements since its conception, such as low cost, low consumption, and high workspace range; finally, it has to perform in outdoor and indoor environments. Therefore, UWB seems to be the most suitable technology due to its high accuracy (10 cm), low complexity, high reliability, low cost, and high detection range signal. Furthermore, UWB is suitable for precision asset location. For those reasons, we have decided to use RF-UWB based devices in our proposed prototype, “2D-CACSET”.

**Figure 3:**
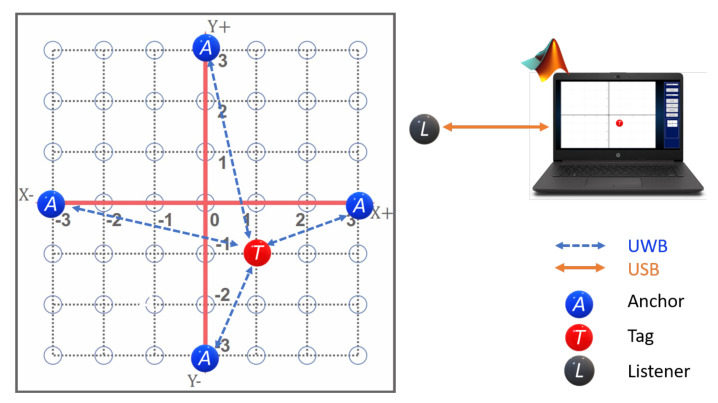
The laboratory involves all three levels in the EMCF-LCM process, physical devices (concrete), symbolic representation (graphical), and interactive practices (abstract).

The proposed prototype for the 2D-CACSET involves the use of a 2D board, a graphical user interface (GUI-2D-CACSET), and three different electronic devices based on the UWB wireless sensor network: Anchors, Tags, and listener, and it is depicted in Figure 3.

It is worth mentioning that we are focusing our efforts on designing and developing an educational kit that enhances learning for industry 4.0 within the EMCF since its conception. This prototype for the 2D-CACSET is devoted to indoor application, considering the classroom as the learning environment. The 2D-CACSET can also be used for outdoor applications.

**Figure 4:**
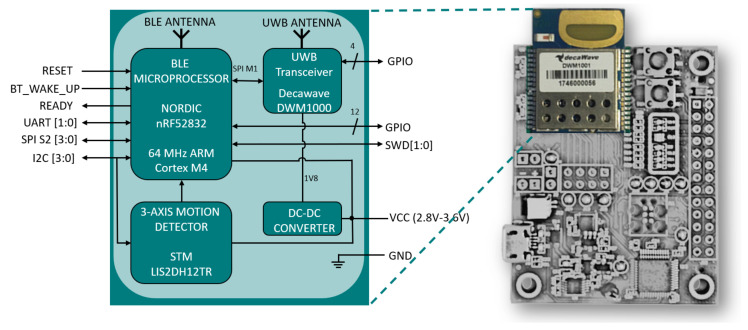
Block diagram of the DWM1001 module (**left**) and the module embedded into a DWM1001-Dev board (**right**).

Before describing each element of the 2D-CACSET, it is crucial to know the UWB wireless sensor and its different network configurations since it is the cornerstone of the toolkit. Inside of UWB Wireless Sensor Network devices, there are DWM1001-DEV boards from Decawave. These development boards are based on the DWM1001 module. The DWM1001 block diagram is shown in Figure 4, and below is the description of each block.

- Microprocessor nRF52832: Ultra-Low Power System on Chip (SoC) integrates Bluetooth Low Energy. Its processor is based on an Advanced RISC Machines (ARM) Cortex-M4 architecture and contains 64 kB of RAM and 512 kB of ROM. An exciting microprocessor feature is that it allows code debugging, which provides a good development environment for programmers and developers.
- DW1000 Ultra-Wideband (UWB) Transceiver: It is the physical layer based on IEEE 802.15. 4-2011, the DW1000 supports two multilateration algorithms, Two Way Ranging (TWR) and Time Differential on Arrival (TDoA), and boasts an accuracy of 10 cm in line-of-sight (LOS) applications. It works in the frequency range of 3.5 GHz to 6.5 GHz. This module is controlled through the Serial Peripheral Interface (SPI). For ease of use, the manufacturer provides a library to control the different peripherals contained in the module.
- LIS2DH12 3-axis linear accelerometer: This I2C accelerometer has a selectable scale range of +/−2 g, +/−4 g, +/−8 g, and +/−16 g at sampling rates from 1 Hz to 5.3 kHz. It bears a self-test capability that allows the user to verify the sensor’s functionality. In addition, the device can be configured to generate interrupt signals when it detects inertial free-fall events and the position of the device itself.
- Step-down converter: This converter provides an output voltage of 1.8, which is required by the DWM1000 transceiver.

The DWM1001 modules [36] integrate a code library called “Positioning and Networking Stack” (PANS), which provides an API that is useful for developers to elaborate their code and algorithms and deploy them on the DW1001 module (PANS Protocol described in Appendix A.1).

**Figure 5:**
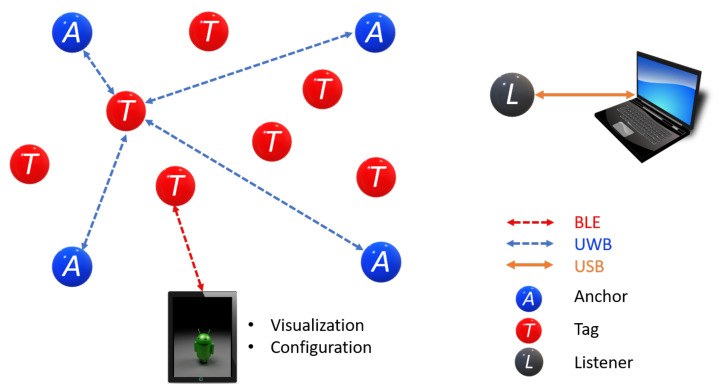
System Configuration Option: 4 Anchors, 7 Tags, and 1 Listener.

As mentioned before, an RTLS is made up of Anchors, Tags, and Gateways. The Anchors are devices with known fixed positions and are responsible for performing multilateration to track a Tag. The Tags are the mobile devices that RTLS tracks. A Listener is an Anchor or a Tagset in passive mode. Therefore, it does not participate in the position determination but has access to all Tags’ information in the network. Finally, a Gateway combines software and hardware that monitors and configures a network through IP protocol in a local area network(LAN)/wide area network (WAN). This work considers a network configuration of four Anchors, seven Tags, and one Listener, as shown in Figure 5. The listener device allows a larger coverage area due to the UWB technology. Furthermore, it admits PC connectivity so that a graphical user interface can be developed, where interactive practices are designed. In addition, the four Anchors can represent each of the axes in the CCS, for an easy setup.

To deploy an RTLS, a network must be created. This network uses a single-sided TWR scheme, in which the Tag ranges with up to four Anchors and then calculates its relative location from the Anchors’ positions, which it has learned from the Beacon messages. The manufacturer provides a graphical interface through an Android application that facilitates external access to the API using BLE. We configured a network with four Anchors, two Tags, and one Listener for the 2D-CACSET. The following subsections describe the wireless network elements.

### 4.1. Anchor

Devices configured as Anchors are devices that must have a known fixed location (x,y,z) coordinates, and through multilateration, calculates the position of a Tag device. Thus, a minimum of three Anchors is required. In addition, one of the Anchors must be configured as an “initiator Anchor”, which synchronizes and controls the network composed of the DWM1001 modules. The Anchor participation in the multilateration protocol is described in Appendix A.1.1.

**Figure 6:**
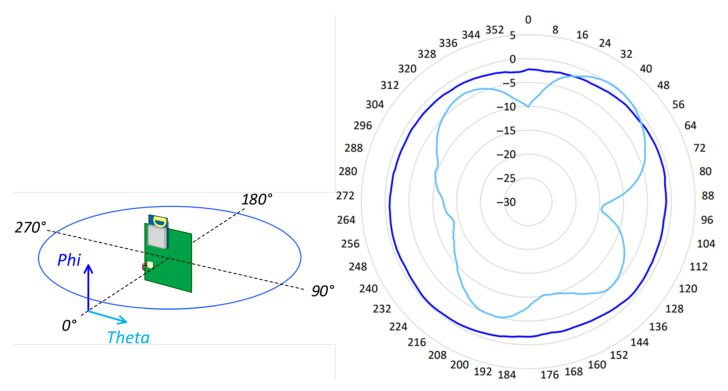
DWM1001-Dev radiation pattern in the *XZ* plane.

According to the datasheet, the DWM1001’s antenna is vertically polarized, which means that the DWM1001-DEV modules are designed to be placed vertically (Referencia). Figure 6 shows the radiation pattern of the omnidirectional antenna in the *X*, *Z* plane when it is observed by another antenna that is also vertically polarized.

The electrical design considers the power supply required to maintain the DWM1001-DEV in continuous operation. The power consumption varies depending on the configuration of the DWM Real-Time Location Systems (DRTLS). Many factors determine the power consumption of the DWM1001-DEV modules, like sampling frequency or transmission gain. According to a study [37], in the case of the Anchors configured with the manufacturer’s default application, the average consumption is 173.29 mA, the Anchors are the devices with the highest consumption since they have to be continuously listening to UWB radio broadcasts waiting for data packets. In our case study, we chose a 3.7v Li-ion battery of 2800 mAh. This battery allows us to have a continuous operation of approximately 16 h. The module has the necessary electronics to charge the battery through a USB port. The following image shows the 3D modeling corresponding to the Anchor.

**Figure 7:**
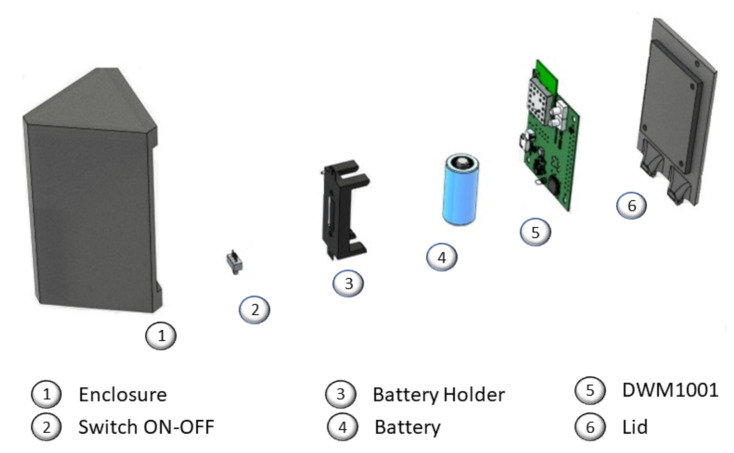
The 3D enclosure for the Anchor device, the enclosure contains all the elements to operate, the elements are listed above.

The mechanical design for the Anchor device is developed using Computer-Aided Design (CAD) software, considering the antenna radiation pattern for better Anchor antenna orientation. The lid of the enclosure has to be colinear to the axis on which it will be placed. In addition, the 3D enclosure keeps the module upright to achieve omnidirectional signal distribution (see Figure 7).

Now, the Anchors’ positions have to be configured through the BLE module using the Android application. The application allows defining coordinates of each Anchor on the network according to the dimensions of the workbench. Table 1 shows Anchors’ coordinates which define the workspace.

### 4.2. Tag

**Figure 8:**
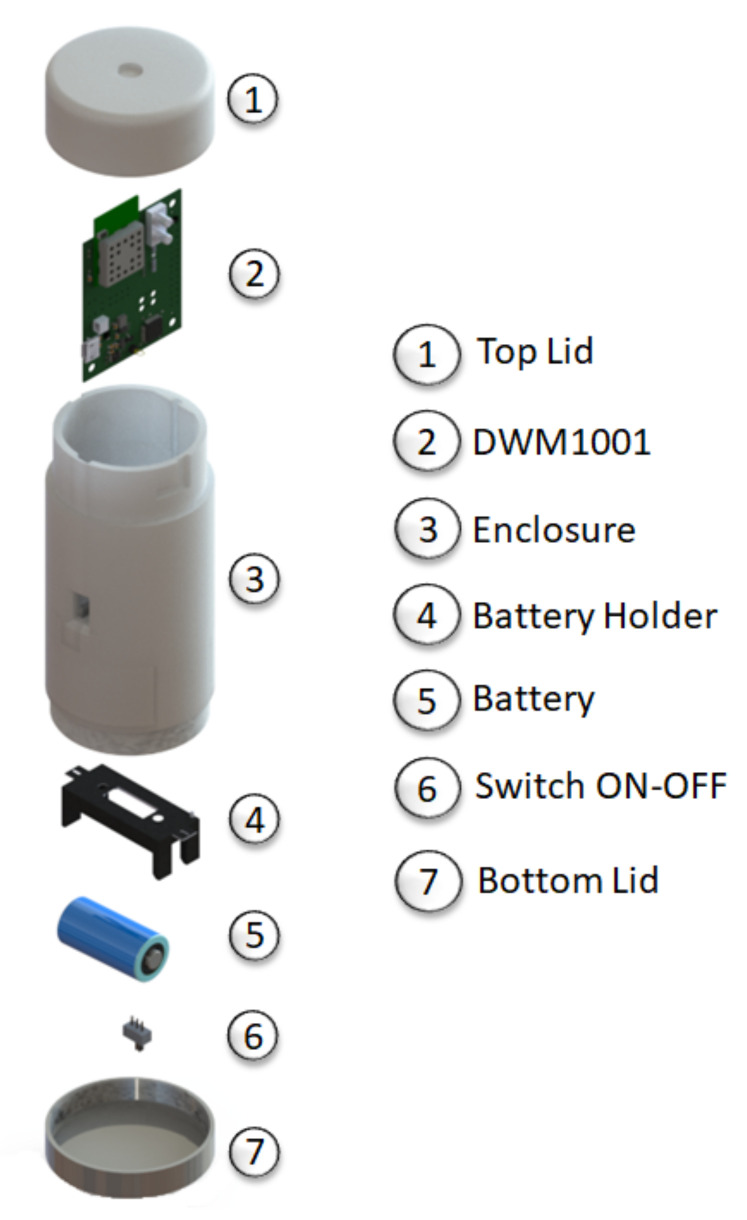
Exploded view of the 3D enclosure for the Tag device, all the elements it contains are listed above.

The devices configured as Tags receive messages from the Anchors using TWR protocol (Appendix A.1.2). The Tag generates a list of Anchors from which it has received information; among the information provided are the 3D coordinates of each Anchor. The Tag chooses, if possible, the four Anchors closest to it, then the Tag calculates its distance to each of the Anchors using the TWR method to obtain its coordinates. As with the Anchor, the electrical design is the same, while the mechanical design must distinguish between them. In this case, a circular shape was chosen; this shape provides students a more ergonomic Tag for easier handling (see Figure 8).

It is worthwhile to mention that, unlike the Anchors, the Tags have lower power consumption because they only participate when they want to calculate their position; this may vary depending on the application. Using the factory firmware, it has an average consumption of 52.84 mA [37], as its enclosure contains a 2800 mAh battery, this would give us a total of 52 h of operation. The module also allows charging the batteries through the USB port.

### 4.3. Listener

The Listener is just a DWM1001 device configured in passive mode, so it does not participate in the position measurement of the Tags; it is not necessary to define its coordinates (more details in Appendix A.1.3). The Listener is designed to access all Tags’ coordinates and to process the data through different means. In our case, the Listener will use this data to feed the training software developed in MatLab© through the USB port. To keep the antenna vertical, we use the same design of the Tag device that we can see in Figure 8, with the only difference that the Listener is of a different color than the Tags.

### 4.4. GUI-2D-CACSET

**Figure 9:**
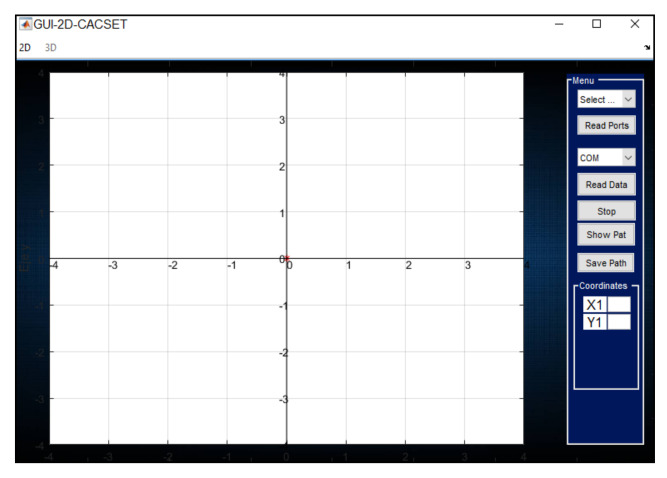
GUI-2D-CACSET developed in MatLab©.

The programming environment chosen for the software development is MatLab© (MathWorks, Inc., Natick, MA, USA), which offers handy mathematical tools and allows to program Graphical User Interfaces (GUI) and connect to different peripherals via UART, Bluetooth, or other communication protocols. A GUI that allows students to interact with the 2D-CACSET, called the GUI-2D-CACSET, was developed for this research. It is depicted in Figure 9.

For the software to work correctly, the listener device and the wireless sensor network must be running. The software has the option “Read Port”, which detects the UART ports present in the equipment. The software allows starting the communication with the listener device. Through the UART API, the AT command ‘LEC’ is sent. This command shows the distances to the ranging Anchors and the position in Comma Separated Values (CSV) format. When a position is successfully calculated, the coordinates are received in the UART port. This information is processed in MatLab© to display it to the user on GUI-2D-CACSET. The software allows selecting between different practices. Depending on which one is selected, different information will be displayed. Three different practices and their corresponding instructional design are described in the following section.

### 4.5. 2D-CACSET Board

**Figure 10:**
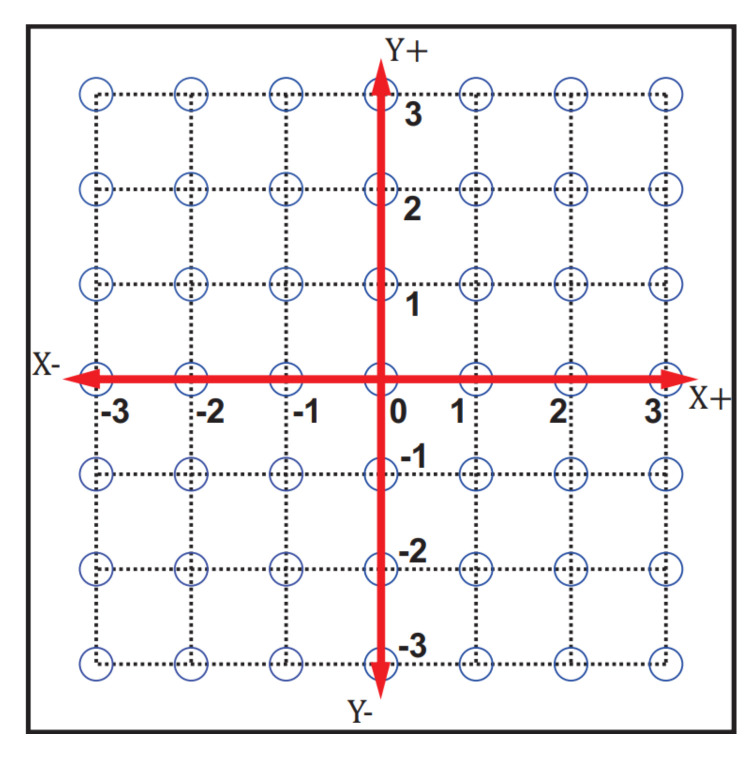
Vinyl design for the Cartesian plane with a grid size of 15 cm.

The 2D-CACSET board, depicted in Figure 10, is a workbench that represents the Cartesian plane in a 2D space area of 110×110 cm and includes the X+, X−, Y+, Y− axes. The workspace of the 2D-CACSET Board is the set of all positions that the Tags can reach and are shown as blue circles. Distance measurement tests were performed to determine the appropriate size of the Cartesian plane grid (see Section 6), which equals 15 cm.

**Figure 11:**
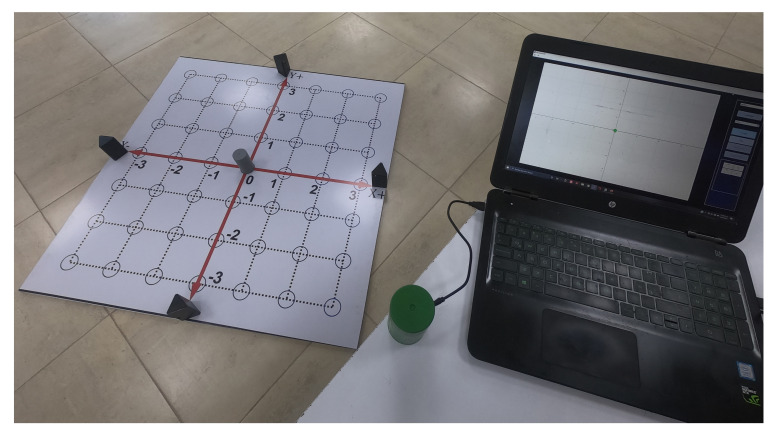
Implemented 2D-CACSET.

The complete 2D-CACSET developed in this work has to start assembling in the following order. First, the 2D-CACSET Board is mounted on a table or the ground. The four Anchors are placed pinpointing X+, X−, Y+, Y− axes of the 2D Cartesian plane in the positions according to Table 1 while the Tags represent the points in the two-dimensional space and can be in any position within the workspace, preferable inside of a blue circle. Finally, a Listening device connected to a PC collects the position information from the Tags; this Listener processes this information and feeds the GUI-2D-CACSET to display the Tag position (see Figure 11).

Finally, the integration of this lab-on-box, called 2D-CACSET, can be embedded within engineering education classes throughout the entire university career. For example, in a typical mechatronic curriculum [38], the lab-on-box can be used in the following classes: calculus, statics and dynamics, vectorial calculus, differential equations, numerical methods, analog control, robotics, sensors and instrumentation, process automation, strengthening workshop upon graduation, among others. The 2D-CACSET can be used from the first semester with the core curriculum courses until more specialized courses. This kit and the instructional design based on the EMCF represent a perfect combination for a learning platform for engineering students. Further information regarding the application of the EMCF refer to [3,11,23].

## 5. Instructional Design for Mechatronic Concept Two-Dimensional Cartesian Coordinates

This research focuses on developing a technological innovation that allows students to improve their higher-order thinking within the educational phase framework using modern technology, specifically mechatronic thinking. This paper focuses on learning the two-dimensional space concept within the EMCF-LCM methodology; including the three learning levels: concrete, graphic and abstract level:

- Concrete Level: This level requires the student to experiment with real-world objects; therefore, the 2D-CACSET represents a way to achieve this. Here the students learn through interaction with artifacts, elements, and devices, depending on the context or discipline. In the spatial context, a bi-dimensional CCS is a widely used way to locate objects in mathematics. Therefore a Cartesian plane is the best choice to start with some basic spatial concepts. For this purpose, the 2D-CACSET board represents the Cartesian plane, and the Tag represents a point in the plane. The student can interact with the Cartesian plane making movements of the Tag; this is done by using colloquial language.
- Graphic Level: In this second level, the student must relate their skills acquired in the first level with symbolic elements. *Software* is a tool that can help at this level. The advantages of any software are that it is widely scalable, expandable, and can be used in a wide range of applications. Developing software also gives us the possibility to generate new capabilities in the future. For our purpose, the software will graphically show the elements that make up a two-dimensional plane and work in conjunction with the physical devices. Thus, an interaction between software and physical devices is done by using the GUI-2D-CACSET.
- Abstract Level: Abstract logical thinking is acquired at the stage of formal operations. Therefore, students can develop a mathematical understanding to enhance cognitive processes without relying on manipulating an object. For this purpose, the software for the graphic level can implement interactive practices, allowing students to strengthen their logical-mathematical intelligence and develop significant learning.

Following the EMCF-LCM methodology, three interactive practices are proposed. Each practice covers the three learning levels reviewed previously.

### 5.1. Practice 1: Cartesian Coordinate System

This practice will help the student understand the concept of coordinate axes. To do this practice the complete 2D-CACSET has to be working with only one Tag.

**Figure 12:**
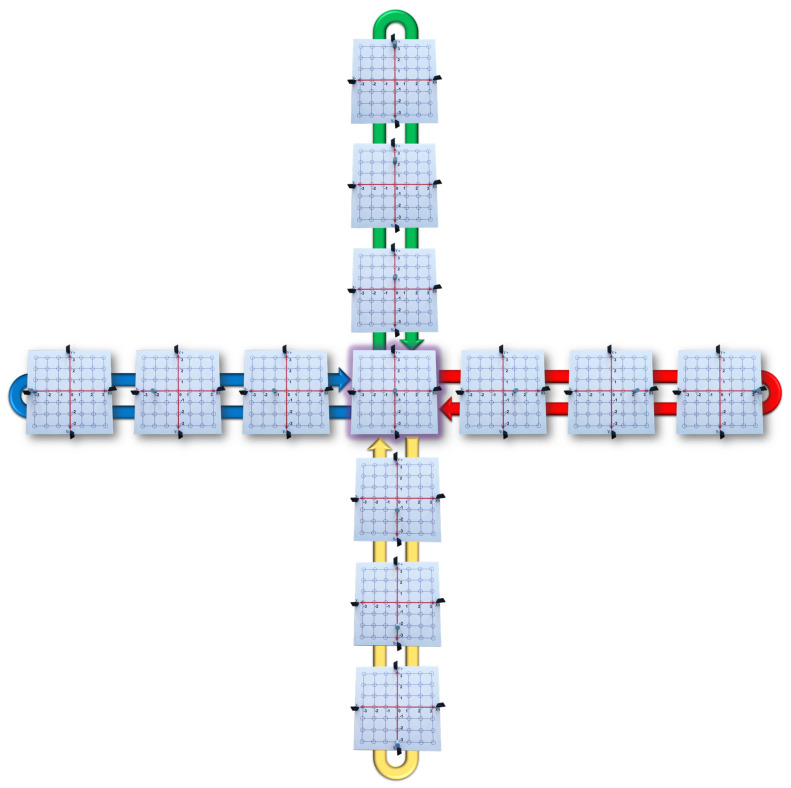
Practice 1: Cartesian Coordinate System.

- Concrete Level:
  1. Move the Tag from the origin to the rightward direction by jumping through each blue circle. Then, return to the origin moving the Tag leftward by jumping through each blue circle (see the red arrow in Figure 12).
  2. Move the Tag from the origin to the leftward direction by jumping through each blue circle. Then, return to the origin moving the Tag rightward by jumping through each blue circle (see the blue arrow in Figure 12).
  3. Move the Tag from the origin to the upward direction by jumping through each blue circle. Then, return to the origin moving the Tag downward by jumping through each blue circle (see the green arrow in Figure 12).
  4. Move the Tag from the origin to the downward direction by jumping through each blue circle. Then, return to the origin moving the Tag upward by jumping through each blue circle (see the yellow arrow in Figure 12).
- Graphic Level:
  5. With a red marker repeat step 1 without separating the marker from the vinyl. Then, assign to this movement the symbol X+.
  6. With a green marker repeat step 2 without separating the marker from the vinyl. Then, assign to this movement the symbol X−.
  7. With a blue marker repeat step 3 without separating the marker from the vinyl. Then, assign to this movement the symbol Y+.
  8. With a yellow marker repeat step 4 without separating the marker from the vinyl. Then, assign to this movement the symbol Y−. Finally, the student obtain a plus shaped draw.
  9. Using the GUI-2D-CACSET and paying attention to the graphical interface repeat steps 1 to 4. Table 2 shows the collected data from the Tag representing the position value of *x* and *y*.
- Abstract Level:
  10. The plus shape is the representation of the 2D CCS.
  11. The horizontal line is know as a *X* axis. It encompasses a set of the real numbers {−3,−2,−1,0,1,2,3}.
  12. The vertical line is know as a *Y* axis. It encompasses a set of the real numbers {−3,−2,−1,0,1,2,3}.
  13. The intersection of the horizontal and vertical line is know as origin, and its representation in 2D coordinates is (0,0) in which the first coordinate correspond tho the *X* axis and the second coordinate to the *Y* axis.
  14. Finally, we can extend this knowledge to assign a pair of coordinate to any blue circle in the 2D-CACSET board to represent a point in the 2D CCS with (x,y) coordinates.

At the end of this practice, the student learned how a point moves along the positives and negatives *X* and *Y* axis and how the 2D CCS is built. The complete practice one can be seen in the following video: https://youtu.be/85Z4_GsmgqU (accessed on 17 September 2021).

### 5.2. Practice 2: Quadrants

Now that we know the 2D CCS, this second practice will help the student understand the concept of the four regions of a 2D plane, called quadrants.

**Figure 13:**
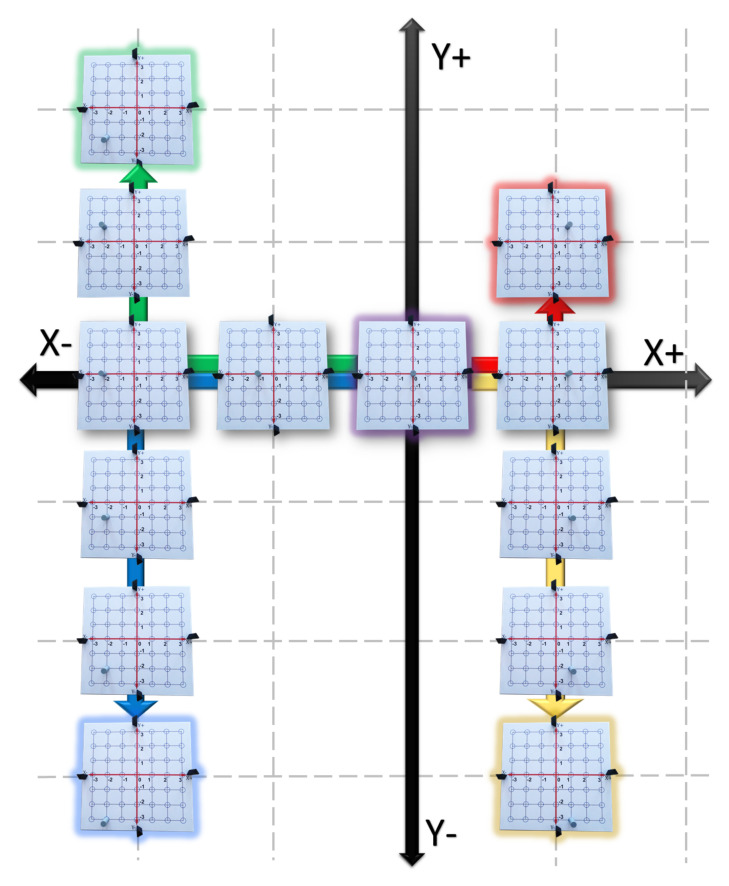
Practice 2: Quadrants.

**Figure 14:**
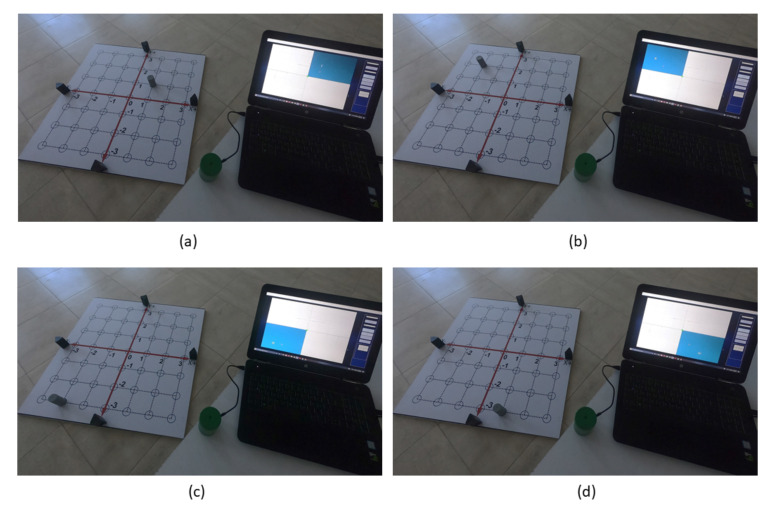
Quadrants displayed in the GUI-2D-CACSET.

- **Concrete Level:**
  1. Move the Tag from the origin to the rightward direction by jumping one blue circle. Then, to the upward direction by jumping one blue circle (see the red arrow in Figure 13).
  2. Move the Tag from the origin to the leftward direction by jumping two blue circles. Then, to the upward direction by jumping two blue circles (see the green arrow in Figure 13).
  3. Move the Tag from the origin to the leftward direction by jumping two blue circles. Then, to the downward direction by jumping three blue circles (see the blue arrow in Figure 13).
  4. Move the Tag from the origin to the rightward direction by jumping one blue circle. Then, to the downward direction by jumping three blue circles (see the yellow arrow in Figure 13).
- **Graphic Level:**
  5. With a red marker repeat step 1 drawing a dotted line in the vinyl and fill in the blue circle you reached. Then, colors the area of the square bounded by the plus shaped that contain the circle. Then, assign to this area the Roman number I.
  6. With a green marker repeat step 2 drawing a dotted line in the vinyl and fill in the blue circle you reached. Then, colors the area of the square bounded by the plus shaped that contain the circle. Then, assign to this area the Roman number II.
  7. With a blue marker repeat step 3 drawing a dotted line in the vinyl and fill in the blue circle you reached. Then, colors the area of the square bounded by the plus shaped that contain the circle. Then, assign to this area the Roman number III.
  8. With a yellow marker repeat step 4 drawing a dotted line in the vinyl and fill in the blue circle you reached. Then, colors the area of the square bounded by the plus shaped that contain the circle. Then, assign to this area the Roman number IV.
  9. Using the GUI-2D-CACSET and paying attention to the graphical interface repeat step 1 to 4 and verify that the illuminated area corresponds to the one that was colored. Table 3 shows the collected data from the Tag representing the position value of (x,y) coordinates. In Figure 14 from (a) to (d) can be seen the Tag position in quadrants II, I, III, IV, respectively.
- **Abstract Level:**
  10. The region labeled with the Roman number I correspond to the quadrant I of the 2D plane. The coordinates in this quadrant I are (+,+).
  11. The region labeled with the Roman number II correspond to the quadrant I of the 2D plane. The coordinates in this quadrant II are (−,+).
  12. The region labeled with the Roman number I correspond to the quadrant III of the 2D plane. The coordinates in this quadrant III are (−,−).
  13. The region labeled with the Roman number I correspond to the quadrant IV of the 2D plane. The coordinates in this quadrant IV are (+,−).

At the end of this practice, the student learned that the coordinate axes divide the plane into four regions called quadrants. These quadrants are numbered counter-clockwise, starting from the upper right quadrant (quadrant I). The complete practice one can be seen in the following video: https://youtu.be/C8zTwAoVId4 (accessed on 17 September 2021).

### 5.3. Practice 3: Point in Cartesian Coordinate System

Now that we know the 2D CCS and its quadrants, this third practice will help the student understand the concept of locating a point in the plane.

**Figure 15:**
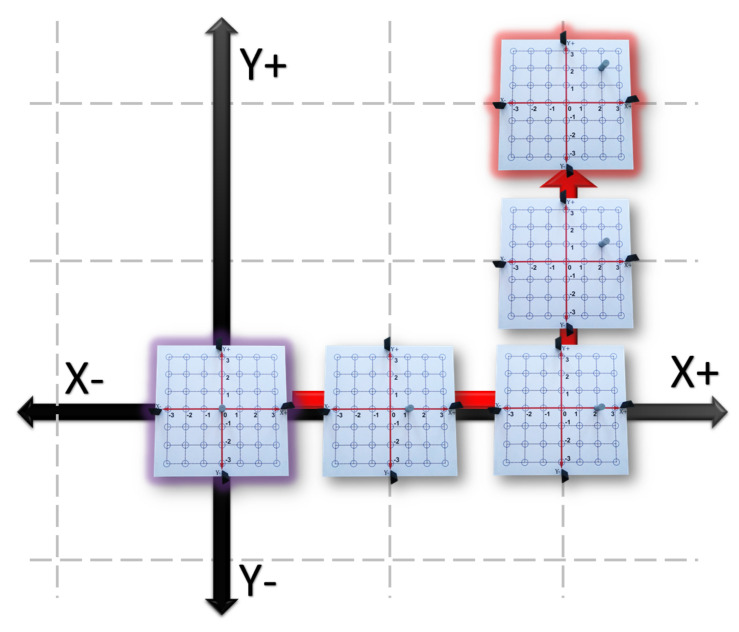
Practice 3: Point in Cartesian Coordinate System.

- Concrete Level:
  1. Move the Tag from the origin to the rightward direction by jumping two blue circles. Then, to the upward direction by jumping two blue circles (see the red arrow in Figure 15).
- Graphic Level:
  2. With a red marker repeat step 1 drawing a dotted line in the vinyl and fill in the blue circle you reached. Then, assign the variable P to this circle.
  3. Using the GUI-2D-CACSET and paying attention to the graphical interface repeat steps 1. Table 4 shows the collected data from the Tag representing the position value of (x,y) coordinates.
- Abstract Level:
  4. The circle you filled can be seen as very small, almost dimensionless. It is perceptible by a contrast of color or relief on a surface and is called a point. The coordinates of a point are two numbers, which are known as an ordered pair when written together. The ordered pair is written in parentheses, with the *x* (also called the abscissa) coordinate and the *y* (or ordinate) coordinate second. The coordinates of point P are (2,2). Finally, we can extend this knowledge to represent any point in the 2D CCS with (x,y) coordinates.

**Figure 16:**
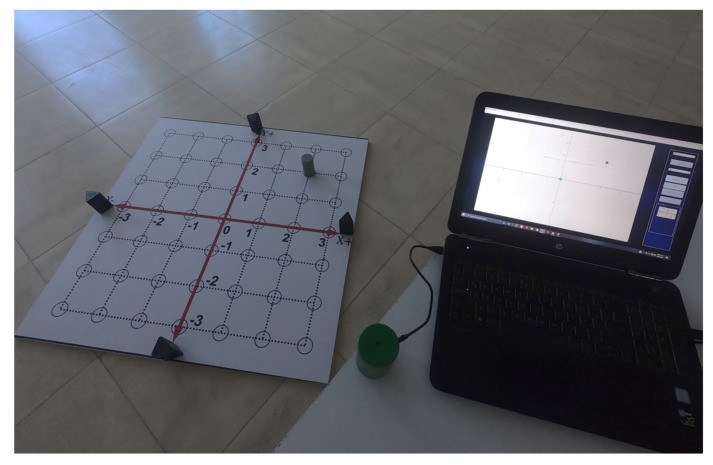
Point P =(2,2) displayed in the GUI-2D-CACSET.

Moreover, the actual displayed position of the point can be seen in Figure 16. At the end of this practice, the student learned the concept of a point in two-dimensional space and its ordered pair of coordinates (abscissa and ordinate). The complete practice one can be seen in the following video: https://youtu.be/GbJZj726WCM (accessed on 17 September 2021).

## 6. Results

**Figure 17:**
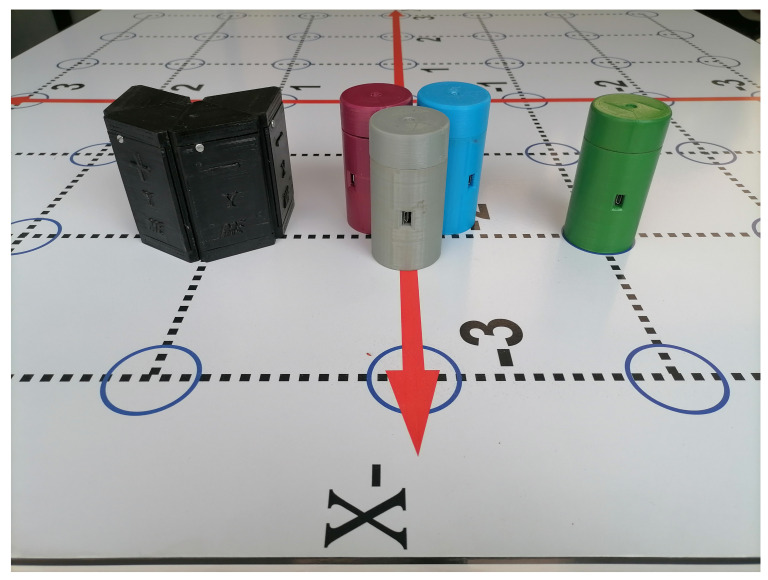
Final version of the Tags, Anchors, and Listener.

One of the main results of this work is the design and implementation of four Anchors, three Tags (gray, red, blue), and one Listener (green) (see Figure 17). The cornerstone of these elements is a real-time location system that uses UWB technology based on the DWM1001 sensor.

**Figure 18:**
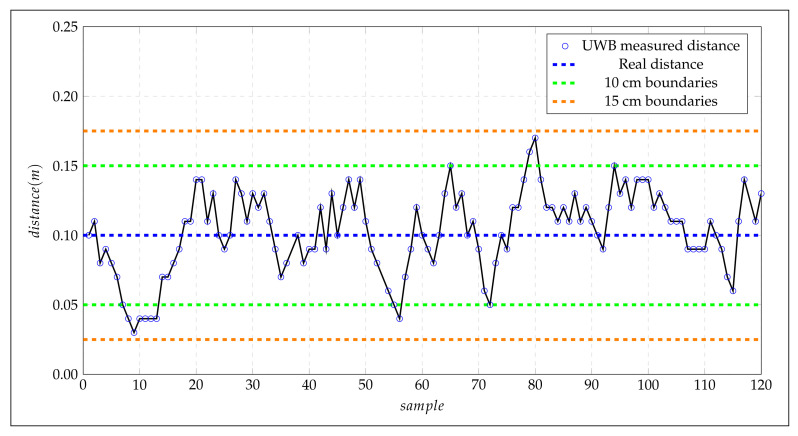
Ranging measurements for Tag positioned in (0,1) coordinates.

Several tests were carried out to evaluate the performance of the DWM1001 sensor. The Tag was positioned in the 2D coordinates (0,1) during measurements, and 120 samples were logged. Figure 18 illustrates the distance measurements samples against actual distance (blue line) for the *Y*-axis. The green and orange lines delimit the accuracy boundaries at 0.1 m and 0.15 m, respectively.

**Figure 19:**
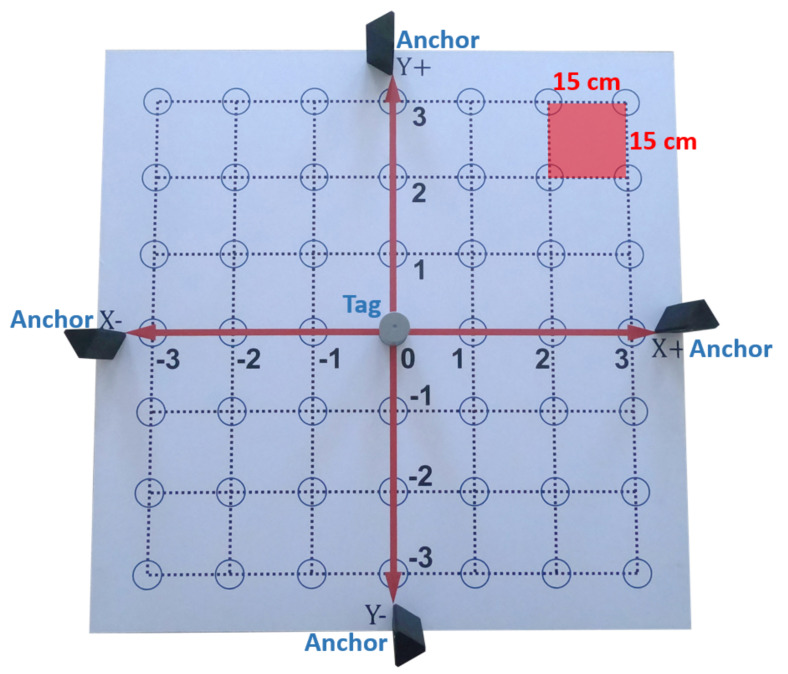
RTLS installed on the 2D board with its grid defined at 0.15 m.

From the measurements, we calculated an average of 0.1036 m and a standard deviation of 0.0290 m; the graph in Figure 18 shows the measurements’ variation in time. It is worthwhile to mention that the manufacturer indicates an accuracy of 0.1 m in the DWM1001 datasheet; however, according to the obtained results, the grid separation on the 2D board is defined in 0.15 m as it is shown in Figure 19.

## 7. Discussion

Technology advancements and new conceptual frameworks have enabled new ways to enhance the learning experience to approach knowledge construction. Educators must take advantage of these advancements to get students from being simple information relayers to knowledge appliers. We presented a novel 2D-CACSET that uses state-of-the-art RTLS technology and EMCF to create a learning platform for engineering students to appropriate several concepts such as the CCS concept and apply it to their further courses.

The two-dimensional Cartesian Coordinates Educational Toolkit: 2D-CACSET was designed and developed following the EMCF guidelines to develop spatial thinking through concrete, graphic, and abstract levels in the student. This approach provides a more robust learning experience than other approaches, like VR, that address only the graphic or abstract level. It also ensures that students appropriate the mechatronic concepts to apply them in totally different environments and more complex situations, going beyond only learning patterns.

The atomic design approach of the workbench and the GUI development of the RTLS-based 2D-CACSET kit using UWB sensor networks allows the creation of instructional designs based on the concept of the Cartesian map. The achieved accuracy is acceptable for the application. Furthermore, the resolution can be adjusted to cover a more extensive area.

The explored concepts in this work are the basic construction elements of the CCS: origin, axes, quadrants, and point coordinates. However, the proposed educational technology allows exploring and expanding its scope to concepts such as distance, angles, polar coordinate systems, and even a 3D coordinate system.

Many students from different areas, such as engineering, did not appropriately grasp the concept of the CCS and still have problems to graph points, lines, vectors, and functions. When these students continue to advance towards their specialty subjects, such as robotics, or control, they face difficulty locating objects in the plane and space.

In principle, this kit is aimed at engineering students but can be applied to basic and upper secondary education students. In the levels prior to higher education, the student does not appropriate or internalize the knowledge about the elements that make up the Cartesian coordinate system and its importance in engineering since this represents the reference frame for more complex mechatronic systems.

**Figure 20:**
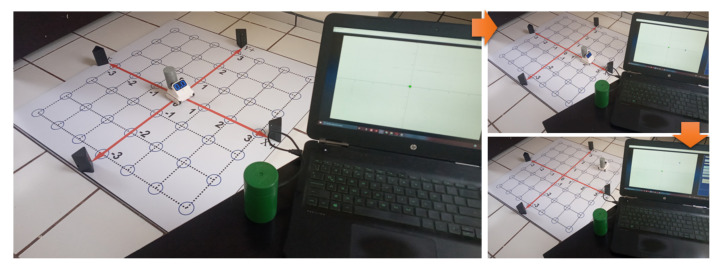
Mobile Robot application for the 2D-CACSET kit.

The 2D-CACSET is an innovative educational tool since it can turn a stage like a classroom into a workspace. It matters not if it is indoors or outdoors. Furthermore, the 2D-CACSET is flexible as different configurations can be loaded in its elements, allowing for new scenarios for more advanced topics or different interaction methods. For example, in a scenario described in this paper, the participant can manipulate the Tag to correlate placement and coordinates. In contrast, for a more advanced scenario, the Tag can be placed on a drone [39] or another mobile robot to carry out trajectory tracking tests (see Figure 20).

**Figure 21:**
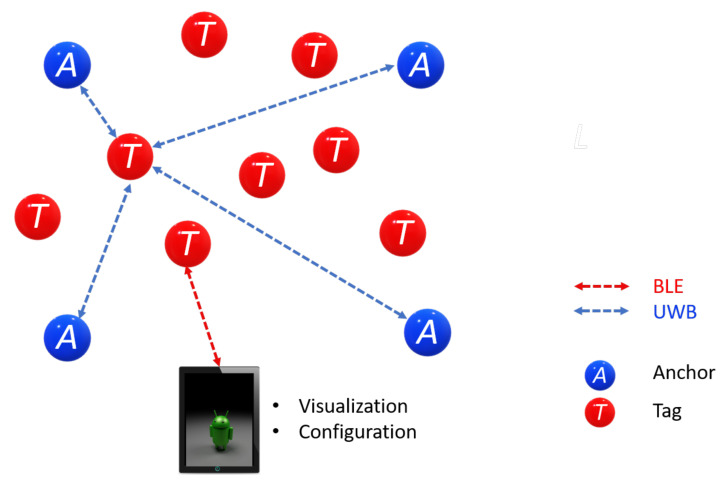
System Configuration Option: 4 Anchors, and 8 Tags.

Some alternative configurations that come from rearranging the RTLS elements are listed next. Four Anchors and eight Tags (see Figure 21). This configuration requires no listener, and two Tag positions can be monitored. This configuration can make up a more restricted kit but is also more economical.

**Figure 22:**
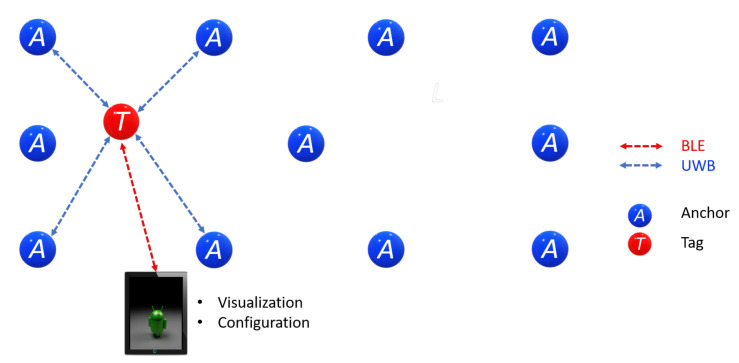
System Configuration Option: 11 Anchors, and 1 Tag.

Eleven Anchors and one Tag (see Figure 22). With a higher Anchors’ density, position accuracy increases. As the Tag selects Anchors dynamically, it can use the nearest Anchor signals and keep accuracy regardless of its position. The downside is a cost increment as more Anchors devices are required. This configuration is recommended for applications where positioning monitoring over large areas is required.

**Figure 23:**
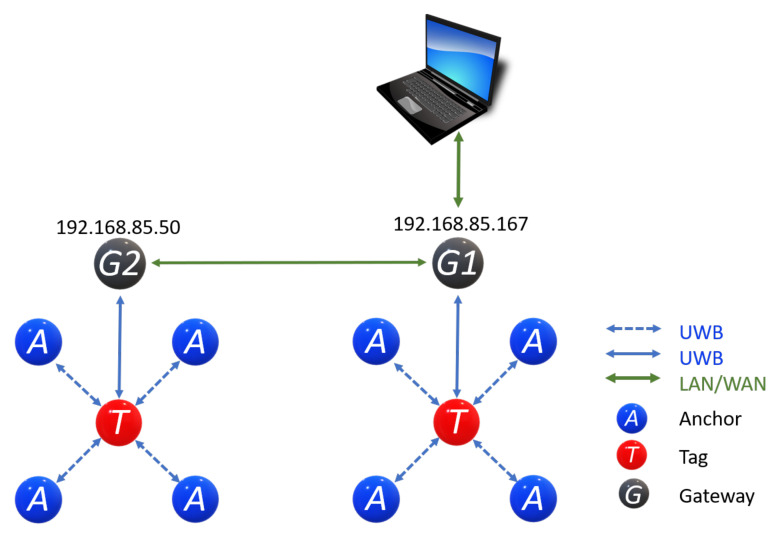
Deployment of Gateway with MDEK1001: 8 Anchors, 2 Tags, and 2 Gateways.

Eight Anchors, two Tags, and two Gateways (see Figure 23). This configuration has as its main advantage that it allows an MQTT server or broker. MQTT enables communication with cloud services, bringing IoT or remote laboratory applications to the table. Nevertheless, this requires more computational resources, increasing cost.

It is important to emphasize that the scalability of the RTLS can be up to 750 Tags, and with an unlimited number of Anchors. However, the system selects the 30 closest Anchors. This feature makes this system a highly flexible tool and opens the range of possibilities to more applications. In summary, using a wireless positioning system based on the UWB system is critical since it offers flexibility to the complete solution of the 2D-CACSET kit in terms of system configuration. For example, the system configuration option four Anchors and eight Tags can be used when a more portable solution is required since the position of the Tags can be visualized on a tablet. Moreover, the 11 Anchors and 1 Tag configuration is helpful to extend the range of the workspace. Finally, the eight Anchors, two Tags, and two Gateways configuration allows us to monitor and visualize several 2D-CACSET kits when working with several classroom teams.

Finally, this learning platform based on the 2D-CACSET and EMCF in Higher Education Institutes could increase their adaptation rate to the evolving concept of Learning Analytics. Then, the students can improve their academic performance and enable teachers to keep track of them, identify students at-risk, and provide timely interventions [40]. Therefore, it is possible to discover hidden patterns in the educational process thanks to the interpretation of student data, e.g., assessing student learning and making predictions, which provides a better understanding of teaching and learning, yielding educational insights.

## Figures and Tables

**Table 1:** Anchors’ coordinates.

Anchor Name	Position (x,y,z) in cm
X+_Anchor	(55,0,0)
X−_Anchor	(−55,0,0)
Y+_Anchor	(0,55,0)
Y−_Anchor	(0,−55,0)

**Table 2:** Positioning collected data in Practice 1.

X+		X−		Y+		Y−	
x Value	y Value	x Value	y Value	x Value	y Value	x Value	y Value
0	0	0	0	0	0	0	0
1	0	−1	0	0	1	0	−1
2	0	−2	0	0	2	0	−2
3	0	−3	0	0	3	0	−3
2	0	−2	0	0	2	0	−2
1	0	−1	0	0	1	0	−1
0	0	0	0	0	0	0	0

**Table 3:** Positioning collected data in Practice 2.

Quadrant 1 (x,y)	Quadrant 2 (x,y)	Quadrant 3 (x,y)	Quadrant 4 (x,y)
(0,0)	(0,0)	(0,0)	(0,0)
(1,0)	(−1,0)	(−1,0)	(1,0)
(1,1)	(−2,0)	(−2,0)	(1,−1)
−	(−2,1)	(−2,−1)	(1,−2)
−	(−2,2)	(−2,−2)	(1,−3)
−	−	(−2,−3)	−

**Table 4:** Positioning collected data in Practice 3.

(x,y)
(0,0)
(1,0)
(2,0)
(2,1)
(2,2)

**Figure A1:**
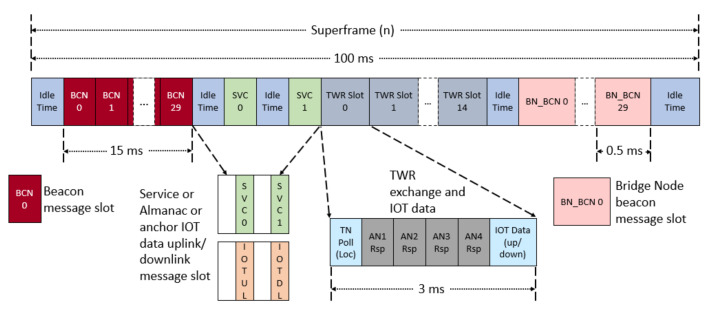
Superframe structure divided into beacon slots, service slots, TWR slot, Bridge node beacon slot, and idle slot.

## Data Availability

Not applicable.

## Abbreviations

The following abbreviations are used in this manuscript:

2D-CACSET	Two-Dimensional Cartesian Coordinate System Educational Toolkit
API	Application Programming Interface
ARM	Advanced RISC Machines
BLE	Bluetooth Low Energy
CAD	Computer Aided Design
CCS	Cartesian Coordinate System
COVID-19	Coronavirus Disease 19
CSV	Comma Separated Values
DRTLS	DWM Real-Time Location Systems
DWM	Decawave Module
EMCF	Educational Mechatronics Conceptual Framework
EMCF-LCM	EMCF Learning Construction Methodology
GPS	Global Positioning System
GUI	Graphical User Interface
IMU	inertial Measurement Unit
IR	Infrared
LAN	Local Area Network
LOS	Line-Of-Sight
PANS	Positioning and Networking Stack
RF	Radio Frequency
RFID	radio frequency identification
RSSI	Received Signal Strength Indicator
RTLS	Real-Time Location Systems
SoC	System on Chip
SPI	Serial Peripheral Interface
STEM	Science, Technology, Engineering and Mathematics
TDoA	Time Differential on Arrival
TWR	Two Way Ranging
UART	Universal Asynchronous Receiver-Transmitter
UWB	Ultra-Wide Band
VR	Virtual Reality
WAN	Wide Area Network
WiFi	Wireless Fidelity

## Appendix A

### Appendix A.1. Decawave PANS Protocol for Location Solution

As mentioned before, the DWM1001-DEV modules are pre-loaded from factory with a firmware called PANS API. This firmware defines the operation of the PANS protocol by which all devices in a network communicate over UWB channels. The protocol defines that all devices in a network must have the same PANID to measure distance. An Anchor must be configured as an initiator Anchor, and it is the one that will take the lead of the communication protocol. At each instant of time, only one pair of Anchor–Tag devices are exchanging messages. The receiving and transmitting timestamp allow determining the distance between them. This type of multilateration technique is known as Two-Way-Ranging (TWR) [41]. A Tag can ping-pong messages with up to four Anchors. This way, it can calculate its relative location from the Anchors.

These messages are called “superframes”, and since RTLS network uses a Time Division Multiple Access (TDMA). Superframes have a defined time width of 100 ms; a superframe can be sent at a maximum frequency of 10 Hz. However, the manufacturer allows choosing between other frequency options (maximum time of 1 min and minimum time of 100 ms). The DWM1001-DEV bandwidth is 500 MHz, and the whole system capacity is 150 Hz, there is defined as the capacity of the network to contain Tags. The number of Tags that can contain a network depends on the transmission frequency. For example, if the Tags are configured at 0.2 Hertz, the network can support up to 750 Tags. In our case, we selected the highest frequency rate allowed by the protocol, which is 10 Hertz. Theoretically, this would allow a maximum capacity of 15 Tags. However, the frequency rate of the Tags may differ between them, depending on the user’s needs. The structure of the superframe can be seen in Figure A1.

Thirty beacon slots that are reserved for Anchors ranging. This does not mean that the system is limited to 30 Anchors, but rather that across the RTLS, no new Anchor can be added. The superframe structure consists of the following:

- Beacon slot: Each superframe starts with thirty Beacon Message slots (BCN), in which Anchors send beacon messages. This means that at most thirty active Anchors can operate in the same area.
- Service slot: Service slots are used for network service and almanacs messages (e.g., network join requests network).
- TWR slot: Two Way Ranging’s 15 slots are used for Tag-Anchor distance measurements, and for uploading/downloading additional data (e.g., IoT sensor data), each TWR slot lasts five milliseconds, in that interval, the Tag can calculate its distance with up to a maximum of 4 Anchors (AN1, AN2, AN3, AN4), It needs a minimum of three Anchors to calculate a location.
- Bridge node beacons slot: There are thirteen bridge node beacons that are used to indicate to Tags if there are any down-link data available for them.
- Idle Time slot: There is guard/idle time at the end of the superframe, which completes the superframe.

#### Appendix A.1.1. 2D CACSET Anchor Operation

The Anchor configured as initiator will start and control the network and keep the clocks of all Anchors synchronized with it to align the superframe timings. Each Anchor will send information (Beacons, service, and almanacs messages) one after another, according to its assigned seat number in the BNC slot. The seat number is assigned according to which Anchors can see the initiator Anchor. Therefore the initiator Anchor is always in slot 0. Initiator Anchor starts an RTLS network by broadcasting Beacons and Almanacs, this information is received for “ordinary” Anchors that join the network, and a seat number is assigned. In our case, three seat numbers are assigned for each Anchor, and once they join the RTLS, they will also send Beacons and Almanacs messages in the assigned slot. The almanac contains the Anchors’ coordinates (specified in Table 1), general information about the network, firmware version, hardware version, etc. The “common” Anchors wait for the beacon and almanac messages. If the ID of the Anchor is found in the almanac information, it responds with polling, which uses TWR slots of the superframe for the messages exchange between Tag and Anchor.

#### Appendix A.1.2. 2D CACSET Tag Operation

The Tag is initially in sleep mode and periodically turns on, waiting for valid beacon messages and almanacs. The Tag keeps turn on for 5 superframes before returning to sleep for a set period. This period can be of 10 s and will extend to 60 s. When the Tag receives the messages, based on the information received, the Tag determines if it is compatible with the firmware, the hardware version, the firmware size, and the network. If it is compatible, the Tag goes on to process the calculation of its position. The Tag selects one of the Anchors in range using the almanac information (from the BNC slot) and places it in a TWR slot. Once four Anchors are selected and put in a TWR slot. It will initiate a location attempt by sending a Broadcasting Group Polling message that contains its location and a list of the four Anchors it wishes to use for ranging. Each Anchor listed in the group poll message will respond by sending messages in turn. The Tag calculates its distance to each of the Anchors and then privately determines its position. We can access the position data through a listener device via UWB.

#### Appendix A.1.3. 2D CACSET Listener Operation

As previously mentioned, the Anchor configured as a listener does not participate in determining the position of a Tag, so its position does not need to be defined. This device is used to access the positions of all Tags in the network. Through external access to the API, we use a USB port to extract the information of the position of the Tags in the network. For this, the API defines some valuable commands to modify parameters in the configuration of the DWM1001 modules. Among the commands, there is one called ’LEC.’ This command allows access to the position information of each of the Tags present in the network. The information is received as follows:

dwm>lec

POS,0,2A14,0.20,0.16,0.80,0x02

The information is in CSV format, and we can access the Tag id, followed by its coordinates in X, Y, and Z, so we use the listener to obtain this information and feed it to the training software.
